# Atherosclerosis in Fabry Disease—A Contemporary Review

**DOI:** 10.3390/jcm10194422

**Published:** 2021-09-27

**Authors:** Ashwin Roy, Hamza Umar, Antonio Ochoa-Ferraro, Adrian Warfield, Nigel Lewis, Tarekegn Geberhiwot, Richard Steeds

**Affiliations:** 1 University Hospitals Birmingham NHS Foundation Trust, Birmingham B15 2TT, UK; hamza.umar@uhb.nhs.uk (H.U.); antonio.ochoa-ferraro@uhb.nhs.uk (A.O.-F.); adrian.warfield@uhb.nhs.uk (A.W.); tarekegn.geberhiwot@uhb.nhs.uk (T.G.); rick.steeds@uhb.nhs.uk (R.S.); 2 Institute of Cardiovascular Sciences, College of Medical and Dental Sciences, University of Birmingham, Birmingham B15 2TT, UK; 3 University of Birmingham Medical School, Birmingham B15 2TT, UK; 4 Sheffield Teaching Hospitals NHS Foundation Trust, Sheffield S10 2JF, UK; Nigel.lewis1@nhs.net; 5 Institute of Metabolism and System Research, College of Medical and Dental Sciences, University of Birmingham, Birmingham B15 2TT, UK

**Keywords:** Fabry, atherosclerosis, ischaemia, perfusion, angina

## Abstract

Fabry disease (FD) is a lysosomal storage disorder characterised by a deficiency in the enzyme α-galactosidase A resulting in sphingolipid deposition which causes progressive cardiac, renal, and cerebral manifestations. The case illustrates a patient with FD who died suddenly, and medical examination demonstrated myocardial scarring and prior infarction. Angina is a frequent symptom in FD. Our own data are consistent with registry data indicating a high prevalence of risk factors for coronary artery disease (CAD) in FD that may accelerate conventional atherosclerosis. Patients with FD also have a higher high-density lipoprotein (HDL)/total cholesterol (T-Chol) ratio which may further accelerate atherosclerosis through expression of early atherosclerotic markers. Patients with FD may develop CAD both via classical atherosclerosis and through formation of thickened fibrocellular intima containing fibroblasts with storage of sphingolipids. Both mechanisms occurring together may accelerate coronary stenosis, as well as alter myocardial blood flow. Our data supports limited data that, although coronary flow may be reduced, the prevalence of epicardial coronary stenosis is low in FD. Microvascular dysfunction and arterial wall stress from sphingolipid deposition may form reactive oxygen species (ROS) and myeloperoxidase (MPO), key atherosclerotic mediators. Reduced myocardial blood flow in FD has also been demonstrated using numerous imaging modalities suggesting perfusion mismatch. This review describes the above mechanisms in detail, highlighting the importance of modifying cardiovascular risk factors in FD patients who likely develop accelerated atherosclerosis compared to the general population.

## 1. Case

**Figure 1:**
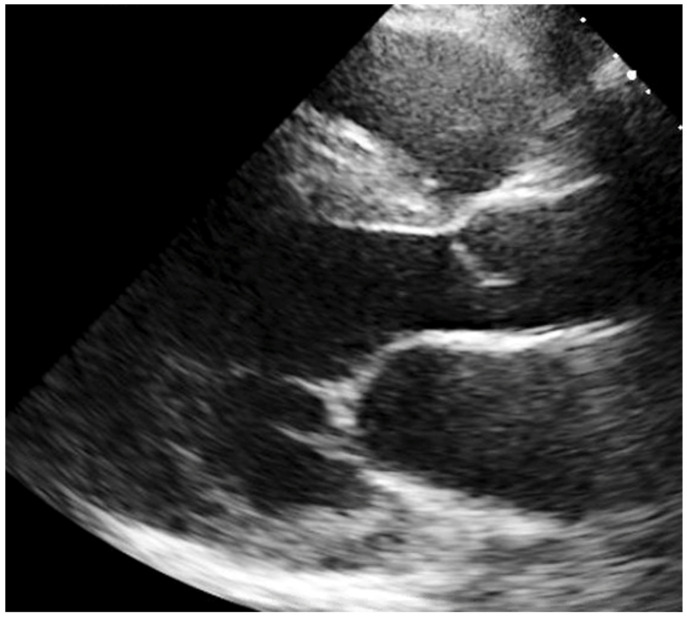
Transthoracic Echocardiogram (TTE) parasternal long axis view demonstrating left ventricular hypertrophy with thinning of the basal inferolateral wall, associated with impaired ventricular function that are typical changes in Fabry disease (FD) cardiomyopathy.

**Figure 2:**
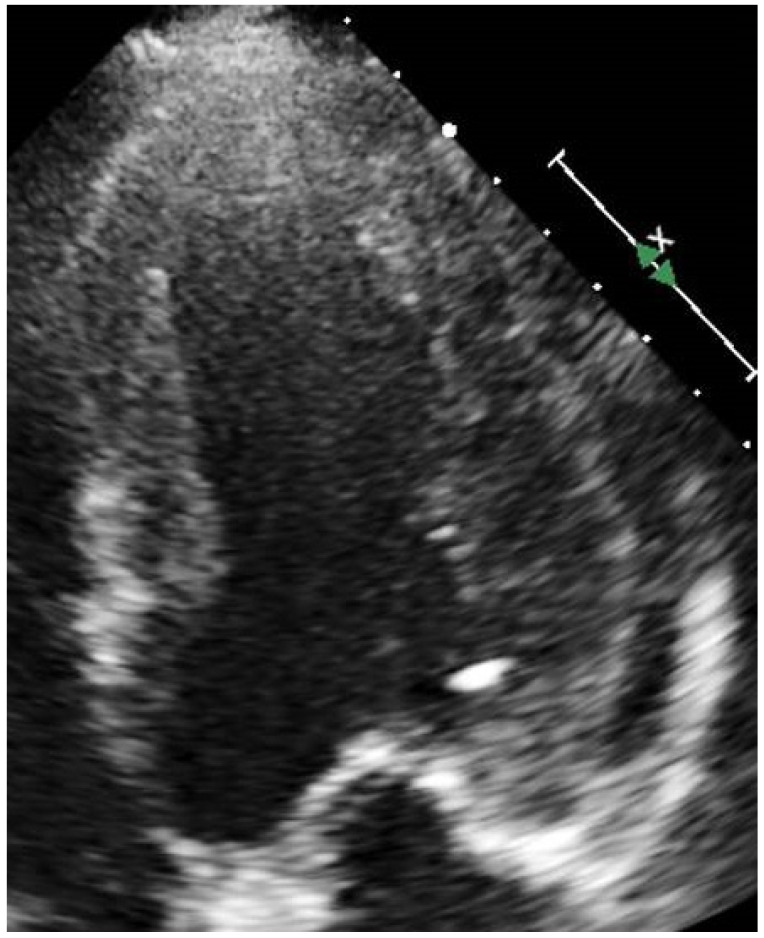
TTE apical 4-chamber view end-systole demonstrating concentric left ventricular hypertrophy (LVH).

A 69-year-old male was seen with a history of chest pain and palpitations. He had a background of Fabry disease (FD) with typical cardiomyopathy phenotype, hypertension, and hypercholesterolaemia. FD was diagnosed in 2012. The patient was hemizygous for the N215S variant mutation. He was first referred to the hypertrophic cardiomyopathy (HCM) clinic with symptoms of angina after a transthoracic echocardiogram (TTE) identified left ventricular hypertrophy (LVH). DNA testing for sarcomeric HCM was negative but alpha galactosidase—A levels were 0.45 µmol/L/hour (normal range 3–20 µmol/L/hour), and Sanger gene testing then confirmed homozygosity for the N215S variant mutation. As well as symptoms of angina, the patient also had pain and paraesthesia in the upper limbs, although nerve conduction studies were normal. In view of his symptoms of angina, he underwent invasive coronary angiography in 2012. This showed moderate mid left anterior descending (LAD) disease and significant ostial circumflex disease which was managed medically (Figure 1 and Figure 2). His resting 12-lead electrocardiogram showed sinus rhythm with first degree atrio-ventricular block and right bundle branch block.

**Figure 3:**
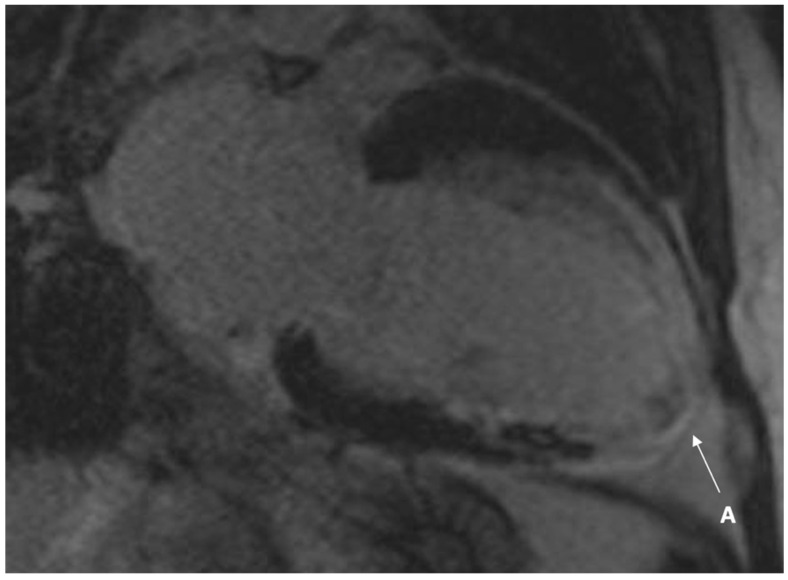
Cardiac magnetic resonance imaging (CMR) vertical long-axis view demonstrating extensive apical transmural late gadolinium enhancement in a region with myocardial thinning and akinesis, consistent with completed myocardial infarction (A).

**Figure 4:**
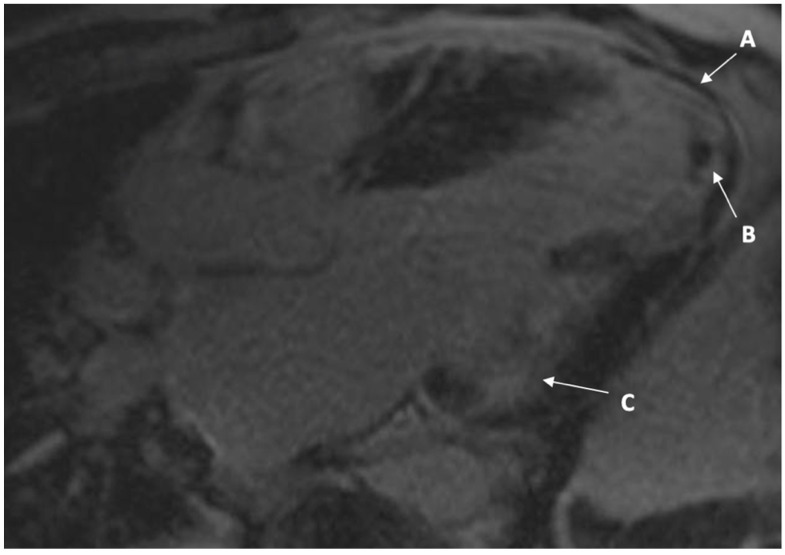
CMR three-chamber view demonstrating extensive apical transmural late gadolinium enhancement in a region with myocardial thinning and akinesis (A), which could be explained by FD with cardiomyopathy but most likely consistent with completed myocardial infarction in the LAD and circumflex territory that was confirmed on histology. In addition, there is an apical thrombus visible (B). There is also the region of thinning in the basal inferolateral wall with late enhancement (C), consistent with typical fibrosis seen in FD.

In 2014, the patient underwent a further TTE which detected impaired left ventricular function with regional wall motion abnormalities and myocardial thinning, with an apical thrombus. Following gadolinium-based contrast on cardiac magnetic resonance imaging (CMR), thrombus was confirmed at the apex, with extensive subendocardial late gadolinium enhancement (LGE). Tissue characterisation with T1 and T2 mapping were not performed at time of presentation, and early enhancement images were not available (Figure 3 and Figure 4). Increased wall thickness on CMR was concordant. He had a cerebral magnetic resonance imaging (MRI) scan which demonstrated scattered white matter changes and 24 h urinary protein confirmed microalbuminuria. The patient was commenced on enzyme replacement therapy (ERT) in 2012 (agalsidase alpha). This was switched briefly to oral chaperone therapy (Migalastat) in 2017 but he was recommenced on ERT in view of side effects (nausea). He was treated with optimal heart failure medications, including an angiotensin converting enzyme (ACE) inhibitor, beta blocker, and mineralocorticoid receptor antagonist (MRA). The ventricular thrombus was managed with anticoagulation in the form of warfarin and aspirin was discontinued.

**Figure 5:**
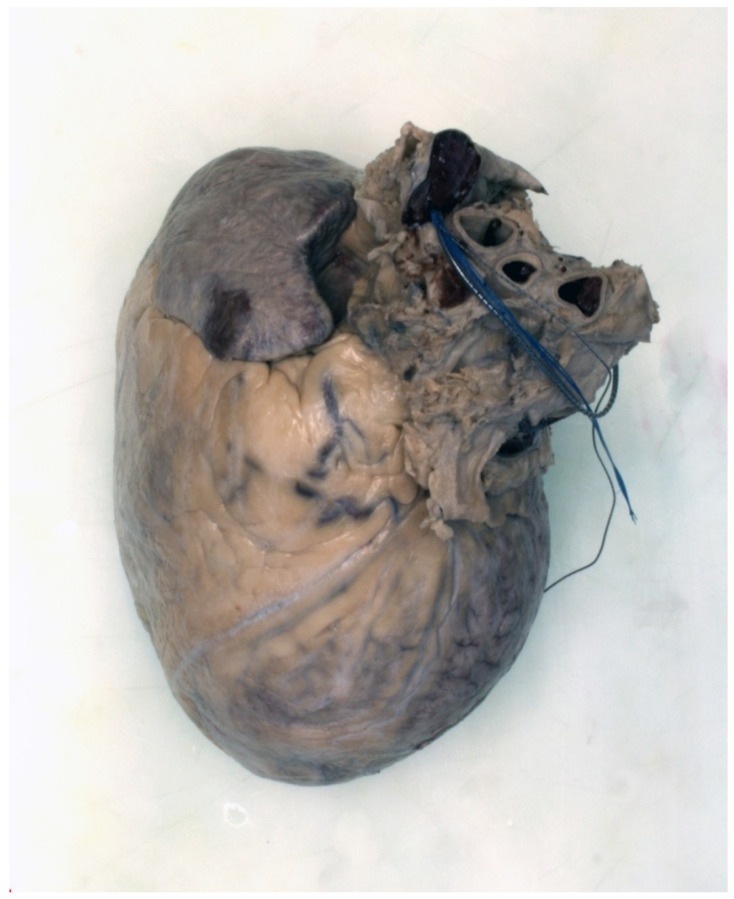
Anterior view of embalmed intact heart prior to dissection—there is global massive cardiomegaly (empty heart weight 950 g). Severed remnants of the ICD wires are visualised around the base of the heart.

**Figure 6:**
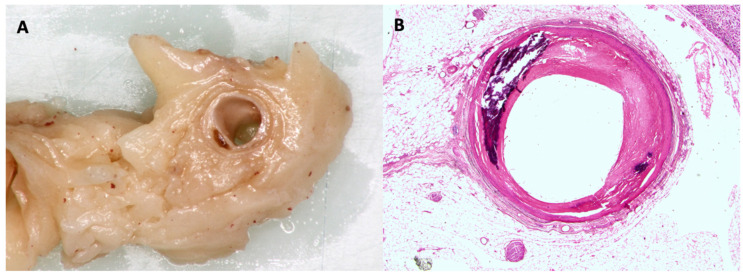
(**A**) Cross sectional slice through mid-right main coronary artery demonstrating eccentric atherosclerotic plaque with localised medial calcification. (**B**) Corresponding histological section of mid right main coronary artery depicting fibro-intimal atheromatous thickening and multi-centric fractured mineralisation of tunica media (haematoxylin and eosin stain; original magnification ×1.25).

**Figure 7:**
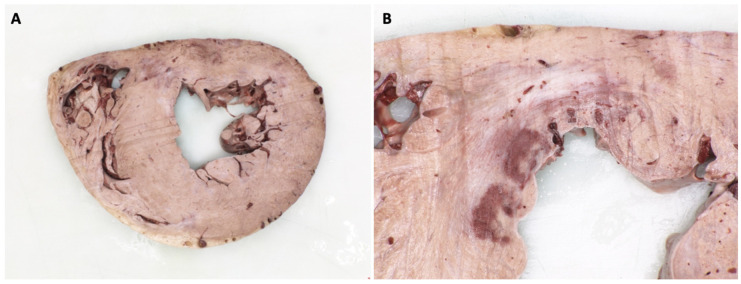
(**A**) Short axis mid-septal ventricular slice through the embalmed heart showing very considerable asymmetrical septum/anterior wall predominant left ventricular hypertrophy (up to 32 mm thick) and right ventricular hypertrophy (up to 8 mm thick). (**B**) Close-up view of healed postero-septal sub-endocardial myocardial infarct scar (the blood clot is an artefact of embalmment; there was no microscopical evidence of more recent acute infarct extension in this region).

**Figure 8:**
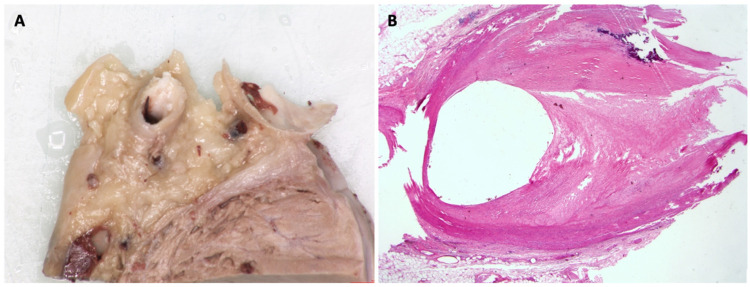
(**A**) Transverse slice through proximal left anterior descending coronary artery illustrating significant luminal stenosis by eccentric atherosclerotic plaque. (**B**) Corresponding histological section through left anterior descending coronary artery showing severe fibrous intimal encroachment into the lumen plus fractured focal calcification (haematoxylin and eosin stain; original magnification ×1.25).

In September 2016, the patient had an acute admission to hospital with sustained ventricular tachycardia (VT). In view of this, he underwent repeat invasive coronary angiography which was similar to that performed in 2012, with persistent, moderate mid-LAD disease and significant ostial Cx disease. A decision was made to implant a dual chamber implantable cardioverter defibrillator (ICD). In 2018, he was admitted following ICD shocks for ventricular arrhythmia without evidence of heart failure, ischaemia, or electrolyte disturbance. Despite anti-arrhythmic medication and VT ablation, he continued to have VT, deteriorated, and subsequently died. Examination of the heart and histological assessment demonstrated chronic myocardial scarring corresponding to previous healed regional myocardial infarction that was consistent with the regional wall motion abnormality in a coronary artery distribution, with myocardial thinning and late enhancement imaging that had been performed pre-mortem (Figure 5, Figure 6, Figure 7 and Figure 8). This case illustrates the point that, as Fabry patients live longer, they are susceptible to acquired heart disease in the same way as the general population.

## 2. Introduction

Fabry disease (FD) is an X-linked multisystem lysosomal storage disorder caused by a deficiency in the enzyme α-galactosidase A [1]. This results in the accumulation of sphingolipids, including globotriaosylceramide (Gb3), and globotriaosylsphingosine (lyso-Gb3) [2]. Progressive accumulation ultimately leads to end-organ damage and subsequent life-threatening renal, cardiac, and cerebrovascular manifestations [3]. Over time, renal failure has been replaced by cardiovascular disease as the most common cause of morbidity and mortality in FD, with relative rates differing according to the source of data and definitions of endpoints. The Fabry Outcomes Survey reported 38% deaths due to cardiovascular disease, compared to 7% attributed to renal disease and 9.5% to cerebrovascular disease [4]. In comparison, the Fabry Registry identified the cause of death to be cardiovascular in 53%, renal in 10.6%, and cerebrovascular in 12% reported cases [5]. Life expectancy in FD is limited to an average 58 years in males and 75 years in females, although 10-year follow-up registry data suggest a modifying effect of enzyme replacement therapy (ERT) on serious organ complications and survival [6,7]. Traditionally, atherosclerotic coronary CAD has been considered an uncommon occurrence in FD, and symptoms such as chest pain and shortness of breath are often attributed to microvascular dysfunction, altered oxygen supply-demand mismatch in left ventricular hypertrophy and reduced arterial compliance. Figure 5, Figure 6, Figure 7 and Figure 8 however, show the post-mortem findings of a 69-year old male diagnosed with FD in 2013 and treated with ERT, which confirmed myocardial infarction as a result of occlusive thrombus complicating atherosclerotic CAD. The aim of this article is to review symptoms, risk factors, and evidence behind the relative risks of atherosclerotic CAD and the disease-specific causes for chest pain, myocardial ischaemia, and death in FD. As patients live longer, clinicians caring for patients with FD need to take into account susceptibility to the risk factors and presentations of cardiovascular morbidity and mortality that are found in the general population.

## 3. Angina

Sphingolipid accumulation can take place in all cardiac cell types leading to left ventricular hypertrophy (LVH), arterial stiffness, conduction abnormalities, and valvular disease [4,8]. Cardiac symptoms are common and include angina, dyspnoea and palpitations. Angina affects between 22–23% patients at a mean age 36–42 years, a frequency that is similar in both genders but with an onset that is typically earlier in males. Symptoms are more common in those patients on treatment than those not on treatment, presumably reflecting more advanced disease and, in particular, greater LVH [9]. Angina is severe enough in these cases to limit quality of life, with the majority affected having greater than or equal to Class II limitation using the Canadian Cardiovascular Society (CCS) grading, which means that the patient develops chest pain on vigorous activity such as walking quickly up a flight of stairs, walking after eating or on a windy day [10]. Prevalence of cardiac symptoms increases with age and although more common in those on ERT, appears to be stable in the majority (26/42; 63%) when on long-term therapy for 10 years or more [9].

Although direct comparisons have not been performed, these data contrast with the occurrence and severity of angina in the general population. For example, the large multinational, multicentre Clarify registry included 32,703 patients with chronic coronary syndrome from 45 countries, of whom 7212 (22.1%) had angina [11]. The mean age of adults with angina was 64.2 + 10.5 year, with the large majority being male (78%) [11]. In those 7212 patients with angina, 29% had only Class I limitation, with the rest having Class II or higher symptoms.

In our case, the patient presenting with angina and was found to have coronary artery disease that was treated medically, at the same time as investigations revealed LVH that was subsequently confirmed due to FD. In summary, angina seems to occur with a similar frequency in FD as those with chronic coronary syndromes in the general population but is more often found in women, impacts on quality of life at an earlier age and with a higher proportion limited by more disabling symptoms. The diagnostic approach in patient with FD and angina should follow conventional methods of assessing patients with chest pain of suspected cardiac origin with the aim to exclude conventional coronary artery disease as a cause of symptoms [12]. In our practice, this involves clinical assessment of the nature of the pain—whether typical, atypical, or non-anginal; consideration of the likelihood of coronary artery disease taking into account risk factor profile; and a preference for use of non-invasive CT coronary angiography with calcium scoring, given the limitations of ischaemia testing in FD [13].

## 4. Conventional Risk Factors

The likelihood of conventional atherosclerosis increases with age, particularly in men [14]. The large majority of major adverse coronary artery events in the general population are explained by the presence of conventional risk for atherosclerosis—beyond increasing age—including hypertension, hypercholesterolaemia, smoking, diabetes, renal impairment, and obesity [15]. Registry data in FD shows that the prevalence of hypertension is 57% in men and 47% in females [16], with hypercholesterolaemia also being frequent at 33% [17]. The prevalence of chronic kidney disease (CKD) in FD can be as high as 42% [18], with registry data showing 26% suffer from CKD stage 3–5, and proteinuria presenting in 44% males and 33% females [19].

Our own data support this frequency of classical risk factors in FD or active treatment thereof (see Table 1) In a retrospective analysis of our Fabry cohort of 47 patients on enzyme replacement therapy (average age 52.4 years; 47% female), 32/47 (68%) were on anti-hypertensive medication, 18/47 (38%) were on a statin, and 12/47 (26%) had a total Cholesterol (T-Chol) > 5 mmol/L. In total, 13/47 (28%) patients had stage 3–5 CKD and 14/47 (30%) with stage 2 CKD. Moreover, 30/47 (63%) had proteinuria defined as an albumin: creatinine ratio (ACR) > 3 mg/mmol. Given the high frequency of conventional risk factors in adults with FD observed in registry and our own data, it is likely that these accelerate atherosclerosis via a conventional pathophysiological process.

An interesting finding is that high levels of high-density lipoprotein cholesterol (HDL-C)/T-Chol ratio have been observed in FD [20,21]. In the general population, this finding has been associated with a lower cardiovascular risk, certainly in comparison to a raised low-density lipoprotein cholesterol (LDL-C)/T-Chol ratio which is associated with higher cardiovascular risk [22]. In FD however, the reverse may be true because a high HDL-C/T-Chol ratio has been linked with high levels of vascular endothelial growth factor (VEGF) and intracellular adhesion molecule-1 (ICAM-1) [23], both markers of early stages of atherosclerosis [24,25]. The same study showed that patients with raised HDL-C/T-Chol ratio and VEGF/ICAM-1 had a greater number of ocular vascular lesions identified on ophthalmic examination (including arteriolar tortuosity, arteriolar narrowing, broadening of the light reflex with minimal arteriolovenous compression in fundic vessels), although no direct examination of the coronary arteries was performed in this study. Of interest, HDL-C level did not change with enzyme replacement therapy [23]. KCa3.1 (calcium-activated potassium channel expressed in vascular endothelial cells) is downregulated with sphingolipid accumulation which causes endothelial dysfunction [26], suggesting that the high HDL-C/T-Chol ratio may be due to endocytosis of LDL-C to the endothelial cells [23] (sphingolipid accumulation may increase LDL-receptor expression [27]).

In our case, the patient was diagnosed with FD at the age of 69 years, following appropriate invasive investigations for CAD given an adverse risk profile, including both hypertension and hypercholesterolaemia. Conventional risk factors for atherosclerosis are common in FD patients. Although different pathological processes may be driving atherosclerosis, arteriosclerosis, and microvascular disease in FD, it seems logical for aggressive risk factor modification though lifestyle and pharmacological therapy to be promoted to minimise cardiovascular risk.

## 5. Histopathology

Endomyocardial biopsies in patients with FD shows the presence of perinuclear vacuoles within cardiac cells representing sphingolipid accumulation. Myocardial fibrosis surrounding severely narrowed intramural coronary arteries was also observed, suggesting this as a primary mechanism for myocardial ischaemia [28]. Hypertrophy of smooth muscle and proliferation of endothelial cells with accumulated sphingolipids may cause small vessel obstruction and subsequent ischaemia. Replacement fibrosis seen on microscopy with cardiomyocyte loss has been shown to be more common in FD patients with angina compared to those without [28].

Classic atherosclerosis is characterised by formation of atheromatous plaques, which are lesions caused by combinations of fibrous tissue and cholesterol-rich lipids. In contrast, case reports and post-mortem findings in FD have hitherto emphasised the formation of a thickened fibrocellular intima, which contain fibroblasts with storage of Gb3, together with fibrosis and calcification of the media [29]. The latter has conventionally been characterised as a different process to the formation of atherosclerotic plaques, and more akin to fibromuscular dysplasia or arteriosclerosis. Furthermore, there is also evidence of narrowing of myocardial capillaries due to GL-3 inclusion bodies that contribute to a unique coronary artery pathology in FD [30]. These data however were acquired from endomyocardial biopsies of FD patients with an average age of 32 years, before conventional atherosclerotic plaque, may be common.

In summary, given the frequency of conventional risk factors in FD patients—considering the extended life expectancy of contemporary, treated patients—it is conceivable that both sphingolipid accumulation and associated fibrosis, as well as classical atherosclerosis may develop and contribute to increased risk in FD. In our case, histology documented typical atherosclerotic plaque, together with evidence of chronic myocardial infarction, alongside classical histological and cellular changes reflecting sphingolipid deposition. The myocardial changes in our case, including both prior myocardial infarction and hypertrophy with fibrosis, could be potential causes of a terminal arrhythmia in our case, although downloads from the ICD device were not available for examination.

## 6. Calcification, Computed Tomography, and Invasive Angiography

The frequency of epicardial coronary stenosis has not been explored in large scale studies of patients with FD. There are published case reports that described the presence of atherosclerotic coronary artery disease in patients with FD [31,32,33], although invasive coronary angiography in single centre studies have tended to demonstrate a low frequency of epicardial stenosis. In a small, single-centre study of 10 male patients with genetically confirmed FD, average age 54 years, and no risk factors for CAD, none were found to have significant epicardial coronary stenosis on invasive coronary angiography (ICA) [34]. This result was replicated in a study of 38 FD patients without conventional risk factors at an average age of 43 years (15 (39%) female; 25 (66%) asymptomatic). None were found to have epicardial coronary stenosis on invasive coronary angiography, although coronary flow was reduced using thrombolysis in myocardial infarction (TIMI) frame count [28]. These studies included patients both younger (aged 69 years) and without the conventional risk factors (hypertension and hypercholesterolaemia) seen in our case.

Our own data relating to computed tomography and invasive coronary angiography are consistent with these findings, following investigation for recent or active symptoms (see Table 2). Within our cohort, 25/47 (53%) patients have had a formal assessment of their coronary arteries having experienced symptoms of chest pain. 12/47 (26%) underwent an ICA and 13/47 (28%) underwent a non-invasive computed tomography coronary angiogram (CTCA). Of those who underwent an ICA, 7/12 (58%) had no flow-limiting coronary artery disease but 3/12 required coronary artery bypass grafting (CABG) and 2 required percutaneous coronary intervention (PCI). Although none of the patients studied by CTCA had flow-limiting or severe coronary artery stenosis, 7/13(54%) had either mild or moderate coronary atheroma. 9/13 (69%) had normal calcium scores with no coronary calcium. In contrast to the two earlier studies, the cohort studied for clinical indications at our centre were mostly male (68%) and were older (average age 60), suggesting that atherosclerotic coronary artery disease should be considered in the differential diagnosis of these patients as they age.

Consistent with this evidence from ICA and CTCA, there is supportive evidence of accelerated atherosclerosis in FD. Autopsy studies of FD patients show plaques that are more concentric with a white discolouration [35]. It is theorised that the microvascular endothelial dysfunction and arterial wall stress from sphingolipid infiltration results in the formation of reactive oxygen species (ROS) [36]. ROS may increase the risk of vascular dysfunction including superimposed atherosclerosis [37]. Myeloperoxidase (MPO) is a peroxidase enzyme secreted by neutrophils during degranulation and is a key component within atherosclerotic plaques [38]. Its presence within plaques is associated with lesion apoptosis, erosion, and rupture. Elevated MPO levels have been observed in patients with FD suggesting this could be a mediator of accelerated atherosclerosis in the FD cohort [39]. Furthermore, in mouse models, α-galactosidase A deficiency was associated with accelerated atherosclerosis due potentially to nitrous oxide (NO) dysregulation. Excess NO accumulated in the atherosclerotic vessels of mice with GLA deficiency may enhance atherogenesis [40]. The high incidence of myocardial infarction, early stroke, and transient ischaemic attack (TIA) in FD suggest a pro-thrombotic state in those with FD [41,42]. Furthermore, due to progressive sphingolipid accumulation within the kidneys, patients with FD often have CKD which in itself further increases thrombotic risk [43]. An interesting observation is the concurrence of FD with the pro-thrombotic Factor V Leiden (FVL) mutation and subsequent heightened risk of thrombosis observed (5-fold higher risk of stroke with FD and FVL) [44].

**Figure 9:**
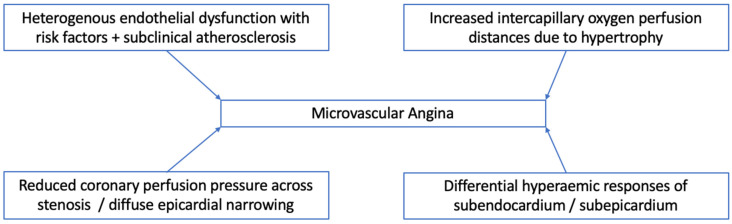
Mechanisms underpinning coronary flow in the absence of angiographic coronary stenosis [46,47,48].

In summary, consistent with case reports of acute coronary events, although atherosclerotic lesions are not an explanation of chest pain in younger, female FD patients, in an older, predominantly male cohort, atheroma that requires revascularisation can be found as in our case. In those without occlusive disease, as in adults from the general population with angina and no evidence of angiographic stenosis, symptoms may be attributed to abnormal coronary flow reserve. Many of these subjects in the general population have been shown to have subclinical coronary atherosclerosis on intravascular ultrasound (IVUS), which has never been performed in FD patients [45]. Mechanisms underpinning coronary flow are multifaceted with varying physiology, and those that may affect patients with FD are highlighted in Figure 9. Supportive evidence of their impact has been found in non-invasive imaging studies.

## 7. Non-Invasive Imaging: Ischaemia

In the study of 38 patients studied using ICA, those with FD exhibited slow flow and slow run off angiographically with delayed opacification of distal vasculature [28]. This slow flow did not correlate with age, gender, or degree of LVH (all patients in the study had LVH). The extent of small vessel disease, however, did correlate with slow coronary flow and myocardial replacement fibrosis. The presence of slow flow in the FD group and absence in control is consistent with microvascular disease and abnormal coronary resistance vessels [49]. In the same study, when exposed to exercise stress, patients with FD developed ST-depression in association with angina. During myocardial perfusion tomography, all patients with angina had an ischaemic response to stress identified by perfusion mismatch.

Abnormal coronary flow in FD was confirmed in a study of 10 adult FD males using gold-standard positron emission tomography (PET) to measure coronary flow reserve (CFR) [34]. Nine patients had symptoms of myocardial ischaemia and underwent ICA to exclude coronary disease as a cause. Interestingly, compared with controls, serum T-Chol and HDL-C was higher in the FD group as has been observed in previously described studies. Resting and hyperaemic CFR and myocardial blood flow (MBF) were both significantly reduced compared with controls with no changes with enzyme replacement therapy. The authors suggested that myocyte hypertrophy and fibrosis secondary to Gb3 deposition could cause increased vascular resistance and subsequent myocardial oxygen demand. This supports the mechanism of microvascular angina due to demand–supply oxygen perfusion mismatch due to hypertrophy in FD (Figure 9). This mechanism is different to that of hypertrophic cardiomyopathy where myocardial ischaemia is usually a result of intramural arteriole remodelling [50,51].

Another study in 10 adults with FD on ERT for a duration of 12 months assessed myocardial perfusion and perfusion reserve using PET [52]. Though coronary disease was not excluded by ICA, none of the patients had angina or signs of ischaemia on ECG or TTE. In all patients, low levels of hyperaemic myocardial blood flow and flow reserve were recorded at baseline and persisted despite treatment with ERT. This supports the mechanism of differing levels of hyperaemia causing microvascular ischaemia in FD.

Work has also been conducted assessing myocardial blood flow (MBF) using multiparametric CMR [53]. In a study of 44 adults with FD, 24 (55%) had LVH, 23 (52%) had evidence of LGE, and 30 (44%) were on treatment (enzyme replacement therapy/oral chaperone therapy). Compared with controls, global stress MBF was lower in FD with no significant differences in rest MBF. Stress MBF was lower when LVH was present but, compared with controls, the LVH-negative cohort had lower stress MBF. These patients had a variety of symptoms (chest pain, breathlessness, and palpitations) which could be explained by reduced stress MBF representing early microvascular dysfunction. The findings suggest that microvascular perfusion abnormalities may precede cardiomyocyte storage and thus be the earliest feature of cardiac involvement in FD. These changes reflect what is observed in studies looking at endomyocardial biopsies of those with FD and angina where endothelial cells were swollen due to sphingolipid storage with arteriolar luminal narrowing due to hypertrophy and fibrosis [28]. The results of this study show that areas with the greatest degree of hypertrophy and sphingolipid deposition (seen as low T1) and fibrosis (high ECV and presence of LGE) are those with lowest MBF. This supports the mechanisms of endothelial dysfunction as a trigger for microvascular angina in FD (see Figure 9). The study also demonstrated greater perfusion abnormalities within the sub-endocardium suggesting that chronic fibrosis predominantly affecting the sub-endocardium over sub-epicardium may result in more perfusion defects in FD patients with LVH.

**Figure 10:**
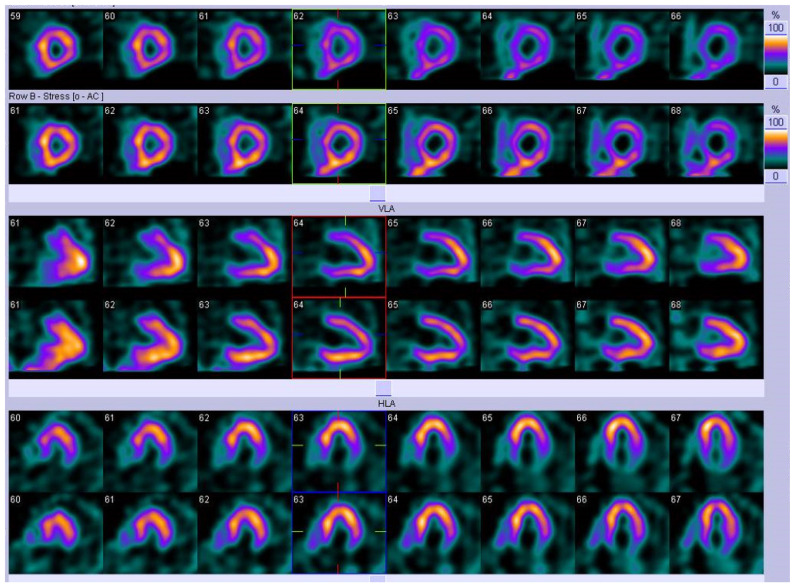
MPS of an adult with FD demonstrating fixed perfusion defects in the basal inferolateral wall of the left ventricle under stress which is typically seen in FD.

In our own cohort of FD patients, five underwent a myocardial perfusion scan (MPS) in view of symptoms of chest pain to assess for perfusion defects. Three of the MPS were normal. One was in a patient with known ischaemic heart disease with a previous CABG. Interestingly the MPS demonstrated fixed perfusion defects in two segments coinciding with basal inferolateral wall LGE on CMR reflecting FD fibrotic change and not ischaemic cardiomyopathy (see Figure 10). The final MPS demonstrated reduced tracer uptake in the anterior wall on stress with improvement at rest in a patient who underwent ICA, demonstrating non-flow limiting disease within the coronary arteries.

## 8. Treatment of Angina in FD

There is no clear evidence that treating symptoms of chest pain or risk factors lowers risk of cardiovascular events in FD patients. There is limited evidence of the effectiveness of conventional angina therapy in FD and future work should aim to explore this in more detail. The use of enzyme replacement therapy has not been shown to improve symptoms of angina or myocardial blood flow after over 12 months of treatment [28,34]. Medical management can be particularly challenging due to the progressive nature of the disease. Added to this, the prevalence of cognitive impairment as well as depression is high in the FD cohort compared with the general population [54] which can affect perception of symptoms and compliance with medications. Arrythmia is a likely cause for many cardiovascular related deaths in FD and ICD implantation is frequent. However, as illustrated in the case, due to the progressive nature of the disease, many patients continue to have sustained arrhythmia that requires advanced treatment. Moreover, the efficacy of some of these therapies including cardiac resynchronisation therapy (CRT) and ICD, do not have an evidence-base in FD.

The main aim of treatment in FD is to minimise end-organ damage. While there is no evidence specifically in FD patients, it is widely accepted that aggressive management of conventional risk factors for atherosclerosis (including lipid lowering therapy, tight control of hypertension and good glycemic control) should be encouraged as well as smoking cessation and regular physical exercise [55]. Aggressive blood pressure control may reduce progression of LVH and use of angiotensin converting (ACE) inhibitors are often used due to renal dysfunction in FD [56]. There is limited evidence that patients with FD who have renal impairment develop accelerated LVH compared to FD patients without renal disease, and that therefore there may be genetic or other modifying factors that work in an incremental fashion on myocyte hypertrophy. Whether this effect is limited to renal dysfunction and whether hypertension is a further modifier of LVH is not known, but it seems reasonable to ensure optimal control of hypertension to minimise risk.

## 9. Conclusions

Angina is a prevalent cardiac symptom in FD and is due to ischaemia that may be secondary to diverse mechanisms, including microvascular disease, altered coronary vasoreactivity, and perfusion mismatch due to sphingolipid deposition within cardiomyocytes and consequent LVH. Ischaemia may also be due to the phenomenon of accelerated atherosclerosis which may be seen in FD and lead to CAD which may result in occlusive thrombus causing myocardial infarction and subsequent death. Patients with FD also demonstrate an increased prevalence of conventional risk factors for CAD and so may develop atherosclerosis though conventional mechanisms as a result of having these risk factors. However, the effect of FD and atherosclerosis is still not completely understood and further research is therefore needed in this area in order to better understand the disease mechanisms involved with the aim of reducing cardiovascular mortality in FD. In patients with FD that have chest pain, whilst this may be due to microvascular dysfunction, it is important to ensure macrovascular CAD is excluded, in particular in the older, male cohort.

## Figures and Tables

**Table 1:** Baseline characteristics of cardiovascular risk factors in a cohort of patients on ERT in a UK-based FD centre.

Characteristic	*N* = 47 (%)
Gender	Male: 25 (53.2) Female: 22 (46.8)
Age (mean)	Male 51.4 Female 53.6 Overall 52.4
Enzyme replacement therapy/oral chaperone therapy	Fabrazyme: 10 (21.3) Migalastat: 20 (42.6) Replagal: 17 (36.1)
Anti-hypertensive medication	0 anti-hypertensives 15 (31.9) 1 anti-hypertensive 20 (42.6) 2 anti-hypertensives 7 (14.9) 3 anti-hypertensives 5 (10.6)
Angiotensin converting enzyme inhibitor/angiotensin receptor blocker	27 (57.4)
Statin therapy	18 (38.3)
T-Chol (mmol/L)	≤5 mmol/L: 35 (74.5) >5 mmol/L: 12 (25.5)
Systolic blood pressure (mmHg)	>140: 16 (34.0) <140: 31 (66.0)
eGFR (mLmin)	>90: 11 (23.4) 60–89: 23 (48.9) 45–59: 10 (21.3) 30–44: 1 (2.1) 15–29: 1 (2.1) <15: 1 (2.1)
ACR (mg/mmol)	<3: 15 (31.9) 3–30: 19 (40.4) >30: 11 (23.4) Not available: 2 (4.3)

Abbreviations: mmol/L—millimoles per litre; eGFR—estimated glomerular filtration rate; mmHg—millimetres of mercury); mL/min—millilitres per minute; mg/mmol—milligrams per millimole; ACR—albumin:creatinine ratio.

**Table 2:** ICA and CTCA findings in adults with FD in a UK-based FD centre including associated cardiovascular risk factors. Abbreviations: mmol/L (millimoles per litre).

Characteristic	ICA *n* = 12 (%)	CTCA *n* = 13 (%)	Total *n* = 25 (%)
Gender/Age	M: 9 (75) F: 3 (25) mean age 65	M: 8 (61.5) F: 5 (38.5) mean age 54	M: 17 (68) F: 8 (32) mean age 60
Angiography findings	Normal: 5 (41.7) Mild coronary disease: 2 (16.6) CABG with patent grafts: 3 (25) PCI with patent stents: 2 (16.6) Significant coronary disease (including occluded grafts/stents): 0 (0)	No coronary stenosis: 6 (46.2) Mild coronary stenosis: 6 (46.2) Moderate coronary stenosis 1(7.6) Significant coronary stenosis 0 (0)	No coronary disease: 11 (44) Mild/moderate coronary disease: 9 (36) Coronary disease with patent grafts/stents: 5 (20) Significant/Severe coronary disease: 0 (0)
Hypertension	5 (41.7)	3 (23.1)	8 (32)
Diabetes	0 (0)	0 (0)	0 (0)
Cholesterol	<4 mmol/L: 7 (58.3) 4–5 mmol/L: 3 (25) >5 mmol/L: 2 (16.7)	<4 mmol/L: 3 (23.1) 4–5 mmol/L: 7 (53.8) >5 mmol/L: 3 (23.1)	<4 mmol/L: 10 (40) 4–5 mmol/L: 10 (40) >5 mmol/L: 5 (10)
Family History	2 (16.7)	2 (15.4)	4 (16)
Smoking History	1 (8.3)	6 (46.2)	7 (28)

## Abbreviations

FD	Fabry disease
CAD	coronary artery disease
HDL	high-density lipoprotein
T-Chol	total cholesterol
ROS	reactive oxygen species
MPO	myeloperoxidase
HCM	hypertrophic cardiomyopathy
TTE	transthoracic echocardiogram
LVH	left ventricular hypertrophy
DNA	deoxyribose nucleic acid
LAD	left anterior descending artery
CMR	cardiac magnetic resonance imaging
LGE	late gadolinium enhancement
MRI	magnetic resonance imaging
ERT	enzyme replacement therapy
ACE	angiotensin converting enzyme
MR	mineralocorticoid receptor antagonist
VT	ventricular tachycardia
ICD	implantable cardioverter defibrillator
ATP	anti-tachycardia pacing
Gb3	globotriaosylceramide
lysoGv3	globotriaosylsphingosine
CCS	Canadian Cardiovascular Society
CT	computed tomography
CKD	chronic kidney disease
ACR	albumin:creatinine ratio
HDL-C	high-density lipoprotein cholesterol
LDL-C	low-density lipoprotein cholesterol
VEGF	vascular endothelial growth factor
ICAM-1	intracellular adhesion molecule 1
ICA	invasive coronary angiography
TIMI	thrombolysis in myocardial infarction
CTCA	computed tomography coronary angiogram
CABG	coronary artery bypass grafting
PCI	percutaneous coronary intervention
GLA	galactosidase alpha
NO	nitrous oxide
TIA	transient ischaemic attack
FVL	factor V Leiden
PET	positron emission tomography
CFR	coronary flow reserve
MBF	myocardial blood flow
ECG	electrocardiogram
ECV	extracellular volume
MPS	myocardial perfusion scan
CRT	cardiac resynchronisation therapy
